# Evolutionary trends in fertility among Chinese women, 1990–2015

**DOI:** 10.1186/s12978-021-01120-z

**Published:** 2021-03-19

**Authors:** Manyu Lan, Yaoqiu Kuang

**Affiliations:** 1 South China University of Technology, 381 Wushan Road, Tianhe District, Guangzhou, 510641 China; 2 Jinan University, 601 Huangpu Dadao Xi, Tianhe District, Guangzhou, 510632 China

**Keywords:** Fertility pattern, Age–period–cohort analysis, Educational attainment, Crowding out effect

## Abstract

**Background:**

Under the one-child policy of birth control, total fertility rates (TFRs) declined rapidly among women in China. TFRs dropped from 2.29 in 1990 to 1.18 in 2010 and to 1.05 in 2015. However, little is known about the evolution of fertility patterns in China during 1990–2015.

**Methods:**

We used population data from 1990 to 2015 and applied age–period–cohort (APC) models to examine temporal changes and used regression models to analyze the effect of education on fertility across periods and cohorts in China.

**Results:**

Age effects assume an inverted U-shaped curve, which increase and then decline across ages, with a peak value in age groups 20–24 or 25–29. Period effects show a U-shaped curve, which first decline and then increase. Cohort effects show an inverted U-shaped plus V-shaped curve, which first increase, then decline and rebound with different age effects and period effects. The APC effect curves of all-order births are similar to those of first birth, but with different magnitudes.

**Conclusions:**

We revealed the evolutionary trends in fertility patterns among Chinese women from 1990 to 2015. The one-child policy exerted a crowding out effect on education. Even if the well-educated women had an intense fertility intention, the fertility policy offset their desire for more children.

## Plain English summary

Women’s fertility rapidly declined under the constraints of the one-child policy in China. The period 1990–2015 is a phase of sustainably low fertility and also affects future demographic trends. However, little is known about the evolution of fertility patterns in China that are attributable to the impacts of age, period, and cohort. In this study, we established models to analyze the evolutionary progress and underlying causal relationships during 1990–2015. Results show temporal changes in fertility over a period of 25 years. Fertility patterns in urban regions are clearly different from those in rural areas. The effect curves of first birth are similar to those of the all-order births, but with different magnitudes, showing remarkable differences not only with respect to age but also period and cohort. The influence of education on fertility is not well reflected under the one-child policy and moreover, the rigid fertility policy exerted a crowding out effect on education. With implementation of the universal two-child policy, the crowding out effect of the fertility policy on education is weakened. Incentives to support this policy should be introduced, to help resolve the issue of high childcare costs. We wish that the birth quota would be abolished in the near future, to help China address the problem of rapid population aging.

## Background

The period 1990–2015 is special in China’s fertility history, representing a sustained era of low fertility. Under the constraints of the one-child policy in China, women’s total fertility rates (TFRs) rapidly declined to below the replacement level (2.1). TFRs dropped from 2.29 in 1990 to 1.18 in 2010 and to 1.05 in 2015. Population aging, shrinking of the labor force, low fertility, and related socioeconomic problems forced the Chinese government to take action. The trend of persistently low fertility forced China to relax its birth control and implemented a universal two-child policy at the end of 2015, allowing nearly all couples to have two children. Still, fertility continues to decline. We wonder if China formed a unique fertility pattern with far-reaching effects during the period 1990–2015, when TFR declined from a level a little below 2.1 to the lowest fertility rates ever recorded. The aim of the paper is to review the historical trend of China’s low fertility and shed light on the formation of this pattern. We used the population data from 1990 to 2015 and applied age–period–cohort (APC) models to examine temporal changes and used regression models to analyze the effect of education on fertility across periods and cohorts in China. The evolutionary trends of Chinese women’s fertility patterns should be considered not only as related to China’s economic, cultural, and policy factors but also within the Chinese historical context on how fertility patterns evolved and what has driven those patterns in the country.

Both fertility level and desired fertility have trended downward since the 1980s, together with increased economic and educational development in China. Moreover, fertility levels in the country have been lower than desired fertility since 1990 [1]. Chinese women’s TFR level during the period 1990–2000 was 1.72–1.76 [2]. Overall, the fertility patterns of urban areas are similar to those of rural areas in China since 1995. In addition, the fertility patterns change similarly in both types of areas [3]. China’s National 1% Population Sampling Survey in 2015 found the lowest total fertility rate ever recorded. Although the proportions of second births triggered by the universal two-child policy increased slightly, but the rate of first birth as the main fertility pattern in China steadily declined. Later childbearing is increasing with time as the contribution made by younger childbearing-age groups is declining. The sustained low fertility has brought a greater focus on the analyses of the evolution of fertility patterns and factor measures.

The evolution of fertility patterns is associated with timing factors. Fertility timing effects are often described as period, cohort, or other temporal variables, e.g., tempo. However, tempo distortions would occur in the calculation of the TFR. Therefore, Bongaarts and Feeney [4] established a tempo-adjusted TFR to eliminate timing effects. The formula is as follows:

1 $${\text{TFR}}_{i} \left( t \right)* = \sum_{i} {\text{TFR}}_{i} \left( t \right)/\left[ {{1} - {\text{r}}_{i} \left( t \right)} \right]$$

where TFR_*i*_(*t*)* denotes the adjusted TFR_*i*_(*t*), and r_*i*_(*t*) represents the rate of change from the beginning to the end of year *t*, in the mean age of childbearing at birth order *i*. Compared with the conventional TFR, the adjusted TFR was considered more helpful to assess fertility trends. Although the Bongaarts–Feeney adjustment is attractively simple, there exist some shortcomings such as unclear conceptual foundation, assumptions that rarely characterize actual populations [5]. It doesn’t solve the tempo-distortion problem [6, 7], even magnifies tempo distortions [5]. In fact, age, period, and cohort are important factors that should be considered in researching the evolution of fertility patterns. The conceptual distinctions of age, period, and cohort effects are important to analyze the fertility trends.

Age effects represent the variations associated with different age groups that are mainly brought about by such events as physical and physiological changes and aging, and that are closely connected to reproductive conditions. Period and cohort effects reflect the influences of social and environmental forces. Period effects represent variations often resulting from shifts in social, cultural, economic, or physical environments or historical events, which influence all age groups, for instance the adjustment of fertility policy. Cohort effects represent variations in fertility across groups of women born in the same year(s) who shared common social background and experienced common historical events, reflecting the effects of early life exposure to socioeconomic, behavioral, and environmental factors that act persistently over time to produce differences in life course outcomes [5, 8, 9].

An important pattern is that women who give birth earlier have more children than those who start late [10, 11]. The regularity of age variations in many social outcomes across time and place reflects the developmental nature of true age changes. However, a weakened, even inverse relationship between age at first birth and fertility occurs in some low-fertility developed countries (e.g., US, Canada) and makes the timing of the first birth lose its importance in the completed fertility [12, 13]. Still, in developing countries such as Ghana, Gyimah [14] found that the effect of age at first birth on fertility has become more important than ever before, regardless of birth cohort.

The cohort perspective on fertility postponement can be traced back to Ryder’s research [15], which focuses on the quantum and tempo effects of fertility based on the changing mean ages of cohort age schedules. However, the cohort perspective has been challenged. Some researchers have shown that the period, not the cohort, is the prime source of variation in fertility behavior [16]. Moreover, period conditions can influence both the timing and the level of cohort reproduction and period can reflect both the timing and the stage level of cohort fertility. The period perspective is valid as well and is essential for studying birth trajectories and the size and age structures of the population [5]. However, period can reflect the timing (or tempo) of fertility but fail to characterize the evolution of fertility (e.g., a postponement of fertility, maybe a later point, but actually no change in cohorts). Only comprehensive estimation to simultaneously account for age, period, and cohort affords a valid approach to summarize the evolutionary trends of fertility. Therefore, APC analysis is suitable for aggregate population data and the impact of historical events.

China has a large population and has undergone a huge demographic transition within such a short time. This means that the period effects and cohort effects would be much more different than those of Western countries. Therefore, according to effect analysis evolutionary trends in fertility could be explained. However, little is known about the evolution of fertility patterns in China that are attributable to the impacts of age, period, and cohort. Owing to identification problem, few demographic studies have succeeded in separately estimating the contributions of age, period, and cohort variation during the evolution of fertility patterns. Few studies have been done to summarize Chinese women’s fertility trends in recent decades, and analysis is lacking of the underlying historical events, which would have an influence on this progress. In this study, period effects, age effects, and cohort effects are analyzed to distinctly uncover the evolutionary trends in Chinese fertility patterns. Within a few decades, China’s fertility policy has stabilized [17] and women have attained much higher educational levels [18], which could influence fertility. Those are important factors worthy of further analysis.

The evolution of fertility patterns is the result of many factors or important events. Education is an important proxy indicator to measure development, also employed in much demographic research [19, 20]. The educational level among women of childbearing age is considered as an important indicator of their socioeconomic status (SES) [21]. Therefore, it is very important to estimate the educational influence on the evolution of fertility patterns. An in-depth understanding of how education relates to fertility patterns requires consideration of temporal variations. Thus, we examined how changes in education levels affect fertility trajectories across periods and cohorts, apart from ages. Since the 1990s, China has paid much more attention to education (e.g., the expansion of higher education since 1999), and women’s educational attainments have greatly improved. The female illiteracy rate was 31.9% in 1990. That number dropped to 13.5% in 2000 and to 7.3% in 2010. In particular, the percentage of illiterate women aged 15–50 years declined to only 1.5% in 2010. The average educational level among Chinese women increased from 5.4 years in 1990 to 7.1 years in 2000 and to 8.4 years in 2010. The percentage of women who completed higher education was 1.0% in 1990 and reached 8.9% in 2010. This percentage is much higher among childbearing-age women. For example, in 2010, 25% of women aged 20–24 years, 20% of those aged 25–29 years, and 14.6% of those aged 30–34 years had attained higher education levels [18]. The progress in education affected fertility greatly, e.g., from 2000 to 2010, as a result of college expansion in Hebei Province, the mean age of fertility increased by 0.3 years, annual births decreased by more than 60,000, and the TFR dropped by 0.1 [22]. The progress in education would be considered as a historical event, which can be measured easily.

The questions arise of which is the greater driving force, the one-child policy or educational expansion, and how do these interact with each other? In this study, we took childbearing-age women as the research sample and used APC models to analyze the evolutionary progress and underlying causal relationships during 1990–2015. We established models to estimate the influence of educational improvement, on the basis of age-, period-, and cohort-effects analysis. Under the constraint of a stable fertility policy, we examined how improvement in women’s educational attainment affected their fertility. We aimed to reveal the temporal patterns embedded within the declining fertility and provide evidence to help researchers and policymakers understand the trajectory shaped by temporal effects.

## Methods

### APC models

APC models were initially introduced by Mason et al. [23] as a general methodology for estimating age, period, and cohort in the fields of sociology and demography. The identification problem induced by the exact linear dependency between age, period, and cohort is a challenge. Some scholars [9, 23–27] have made important contributions toward finding solutions for that problem. Here, we adopted the intrinsic estimator (IE) method, further developed by Yang and Land [27], to estimate APC models. The IE for log-linear APC accounting models [25] can satisfy the criteria for judging the acceptability and utility of APC analysis, which pass both empirical and simulation tests of validity and provide a useful method for estimating the distinct effects of age, period, and cohort.

The basic APC model is written as follows:

2 $$F = a + \alpha A + \beta P + \gamma C + \varepsilon$$

where *F* is the age-specific fertility rate (ASFR), *a* is a constant term, *A* is the effect of age group, *P* is the effect of the survey period, *C* is the effect of the birth cohort, and *ε* is the disturbance term.

We used a Poisson log-linear model to analyze the evolutionary trends in Chinese fertility patterns.

3 $$E(F_{ij} ) = E(\frac{{B_{ij} }}{{W_{ij} }}) = \lambda = \exp (\mu + \alpha_{i} + \beta_{j} + \gamma_{k} )$$

Fertility *F* is assumed to be distributed as a Poisson variate. i.e., $F_{ij} \sim Poisson(\lambda )$. Here, $\lambda$ denotes the expected number of children, and $E(F_{ij} )$ the expected fertility in age group *i* (*i*_*1*_ = 15–19, *i*_*2*_ = 20–24,……, *i*_*7*_ = 45–49) in the *j*th year (*j*_*1*_ = 1990, *j*_*2*_ = 1995,……, *j*_*6*_ = 2015).$B_{ij}$ and $W_{ij}$ respectively denote the number of live births among women in age group *i* and the average number of women in age group *i* in the *j*th year.$\mu$ denotes intercept, $\alpha_{i}$ denotes the age effect for the *i*th age group,$\beta_{j}$ denotes the period effect for the *j*th year, and $\gamma_{k}$ denotes the cohort effect for the *k*th cohort. One of each of three coefficients is set to zero as the reference age, period, or cohort category against which the estimated coefficients for other categories can be compared [26]. Parameterization is done to center parameters such that their sum is zero:$\sum\nolimits_{i} {\alpha_{i} } = \sum\nolimits_{j} {\beta_{j} } = \sum\nolimits_{k} {\gamma_{k} } = 0$. The log-linear Poisson regression coefficients, standard errors, and model fit were calculated using Stata statistical software (StataCorp LLC, College Station, TX, USA).

### Regression models

We established regression models to estimate educational effects on the evolution of fertility patterns, controlling for the variables age, policy, period, and region.

4 $$Fertility = a + \alpha Age + \beta Age^{2} + \chi Edu + \delta Z + \varepsilon$$

where, the explained variable *Fertility* is the ASFR (‰). The independent variables are as follows: *Age* is the age of childbearing women and its squared term (*Age* squared) is mainly used to capture the possible nonlinear effects of age on fertility. Educational attainment qualifications for *Edu* were disaggregated into seven levels: illiterate (less than a primary school education completed), primary school completed, junior high school completed, senior high school completed, junior college or post-secondary certificate completed, undergraduate university education completed, and graduate-level education completed. *Edu* was measured using average years of education (as constructed by Barro and Lee [28]) and set to 3, 6, 9, 12, 15, 16, and 19 years. As an illiterate person may acquire knowledge through social learning (interpersonal communication) and observation, we evaluated this level as 3, rather than 0 years. *Z* represents other control variables, including fertility policy implementation *Pol* measured using the proportion of out-of-quota births (i.e., the proportion of women with more than one child) in the statistical years. *Period* is the period dummy (here, *P*_*90*_ for 1990, *P*_*95*_ for 1995, accordingly. *P*_*00*_, *P*_*05*_, *P*_*10*_, and *P*_*15*_, respectively, for 2000, 2005, 2010, and 2015. We take 1990 as the reference year). *a* is the intercept. *Zone* is the dummy for region, including cities, towns, and villages (here, we take cities as reference). *α*, *β*, *χ*, and *δ* are coefficient terms, and *ε *is the error term.

### Data

Data on fertility used in this study are obtained from the National Population Censuses in 1990, 2000, and 2010, and the 1% National Population Sample Surveys in 1995, 2005, and 2015 in China. China has experienced a long period of low fertility since the 1990s. During that period 1990–2015, the fertility policy (i.e., mostly the one-child policy) has been rigorously implemented, which ensures data continuity and helps in exploring the evolution of fertility patterns among Chinese women. In the following analysis, we disaggregated women of childbearing age (aged 15–64 years old) into seven 5-year age groups: 15–19, 20–24, 25–29, 30–34, 35–39, 40–44, and 45–49 years. 764,717,850 individuals were involved in sampling which were grouped into 168 observation data. We used six periods and 12 successive, 5-year birth cohorts labelled according to their central birth years, to analyze fertility behaviors. To precisely investigate rural–urban variations, we divided the regions into four types: the whole country/national, cities/urban, villages/rural, and towns. We reported the results separately.

## Results

### Descriptive analysis

**Fig. 1:**
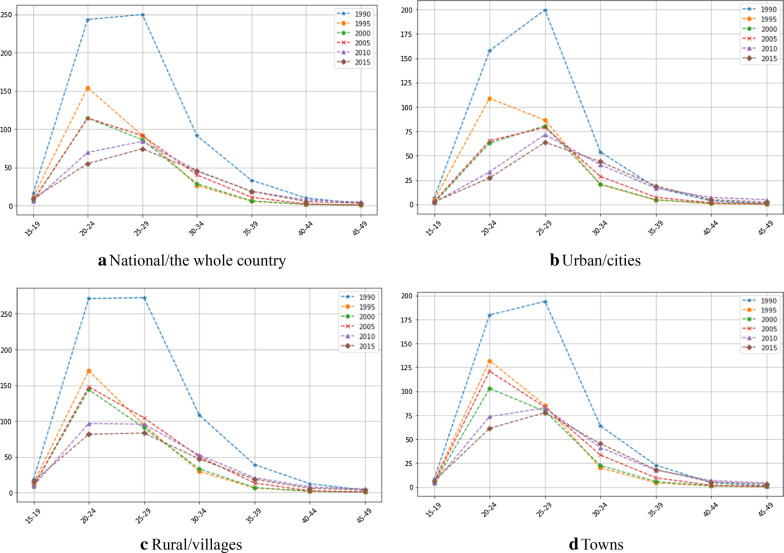
Age-specific fertility rates in urban and rural areas of China, 1990–2015 (the horizontal lines: age; the vertical lines: ASFR (‰))

Figure 1 shows ASFRs by 5-year age-time intervals in urban and rural areas of China during 1990–2015. Fertility declined continuously. The downward trend for women aged 15–19, 20–24, and 25–29 years continued in the whole country. Fertility peaked among women aged 25–29 years. Fertility patterns in urban and rural regions are obviously different. Compared with that in villages and towns, fertility in cities was later and shorter, and the fertile peak ages were further delayed from ages 20–24 to 25–29 years, with fertility curves transiting from an inverted U to an inverted V. However, late childbirth was prolonged, with fertility rebounding among women aged 30–49 years. This is more obvious for women aged 30–34 and 35–39 years since the 2000s. These phenomena have been monitored in 28 European countries since the 2000s where fertility among women aged 30–39 years has rebounded, mainly owing to increased first-birth fertility [29, 30]. Among low-fertility countries, some controversy exists regarding the rise in lifetime fertility rates. However, the increase in the first-birth fertility of the older childbearing-age women reveals that women’s fertility could be delayed in earlier life course, but this delay would be made up for in their later periods. Moreover, there are obvious period effects and regional differences observable in the descriptive data and figures.

**Fig. 2:**
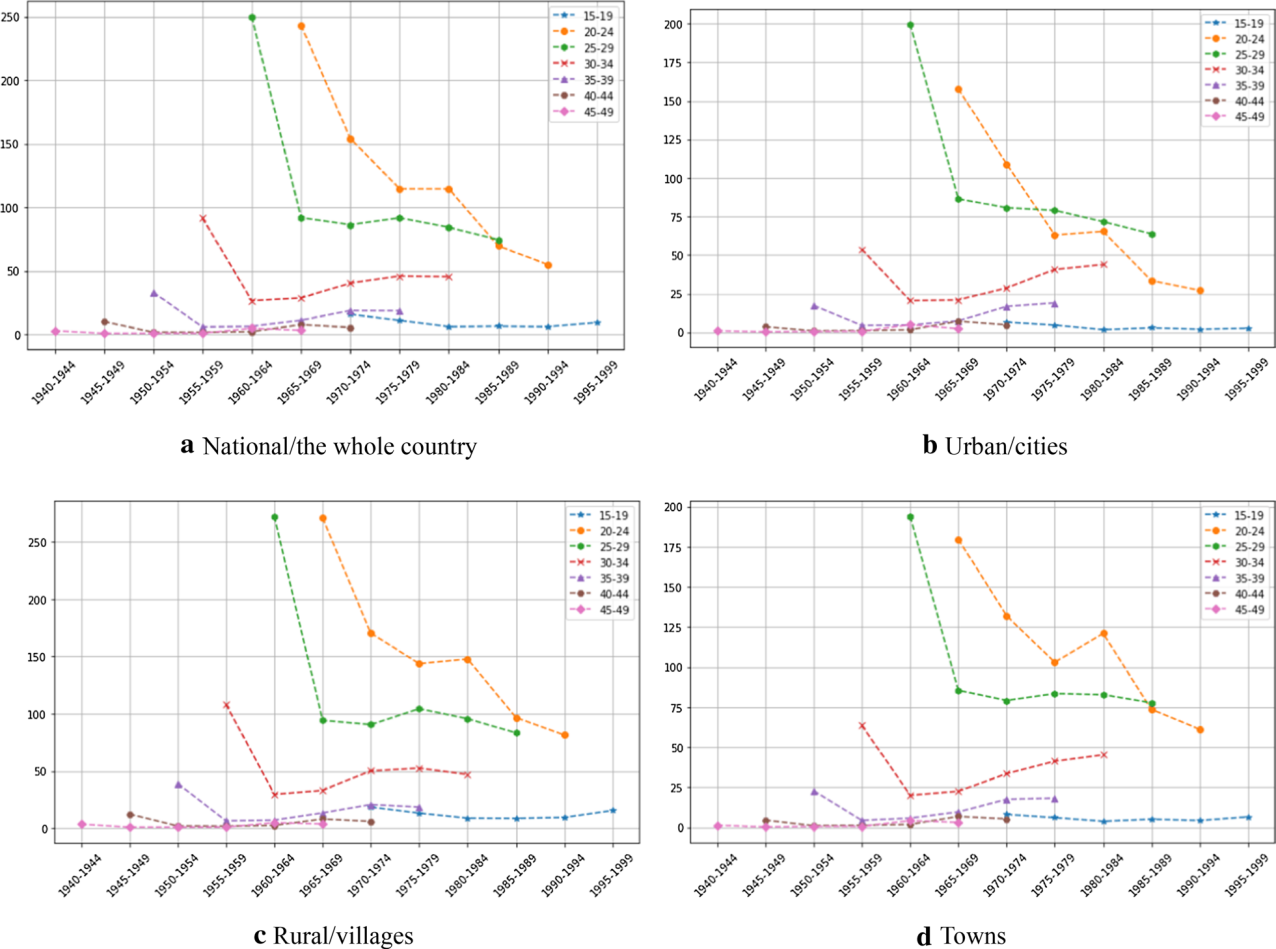
Cohort changes of age-specific fertility rates in urban and rural areas of China, 1990–2015 (the horizontal lines: cohorts; the vertical lines: ASFR (‰))

Figure 2 shows the changes in ASFRs among different regions and different cohorts. Overall, ASFRs declined with birth cohort in an L shape, accompanied by fluctuations, typically in age groups 20–24 and 25–29. The ASFRs in age groups 15–19, 40–44, and 45–49 are relatively stable and level. The ASFRs in age groups 30–34 and 35–39 changed but not obviously. Age effects changed with birth cohorts.

### Intrinsic estimates of age, period, and cohort effects on fertility patterns

**Fig. 3:**
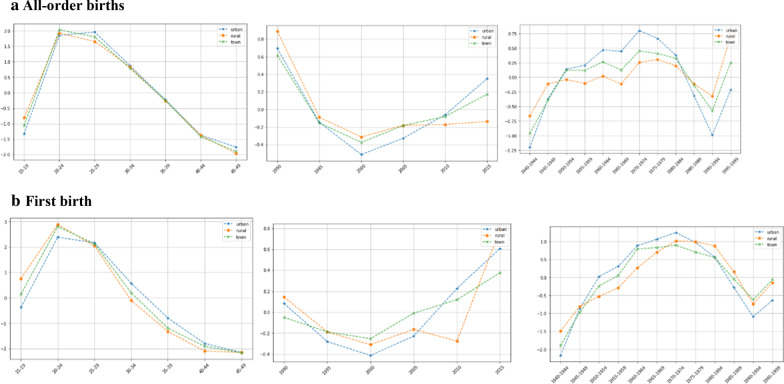
Age, period, and cohort effects (APC analysis)

The estimation results of the APC models using the IE method for fertility are reported separately according to all-order births and first birth in the different regions (Table 1). We first estimated the APC effects of the national all-order births samples and then analyzed regional variations. Effect variations between the all-order births and the first birth were compared accordingly. The effect curves of all three coefficients are shown in Fig. 3.

**Table 1:** Results of estimation of age–period–cohort using the intrinsic estimator method

	National		Urban		Rural		Town	
	All-order	First	All-order	First	All-order	First	All-order	First
*Age*								
15–19	− 0.9204 (0.1402)	0.4394 (0.4037)	− 1.3308 (0.2279)	− 0.3720 (0.5273)	− 0.8096 (0.1234)	0.7491 (0.3861)	− 1.0607 (0.1839)	0.1453 (0.4841)
20–24	1.8830 (0.0703)	2.6871 (0.2653)	1.8502 (0.1065)	2.3802 (0.3375)	1.9253 (0.0637)	2.8837 (0.2573)	2.0327 (0.0890)	2.8018(0.3148)
25–29	1.7070 (0.0695)	1.9101 (0.1607)	1.9599 (0.0864)	2.1622 (0.1886)	1.6547 (0.0652)	2.0403 (0.1644)	1.8046 (0.0776)	2.1214(0.1828)
30–34	0.8208 (0.0887)	0.1651 (0.1588)	0.8748 (0.0997)	0.5717 (0.1363)	0.8338 (0.0838)	− 0.1059 (0.1900)	0.7905 (0.0947)	0.1978(0.1662)
35–39	− 0.2471 (0.1265)	− 1.1681 (0.2874)	− 0.2257 (0.1444)	− 0.7965 (0.2672)	− 0.2634 (0.1198)	− 1.3246 (0.3281)	− 0.2468 (0.1355)	− 1.1951 (0.3151)
40–44	− 1.3539 (0.1869)	− 1.9806 (0.4432)	− 1.3718 (0.2217)	− 1.7947 (0.4477)	− 1.3730 (0.1762)	− 2.0930 (0.4878)	− 1.4229 (0.2092)	− 1.9073 (0.4777)
45–49	− 1.8895 (0.2498)	− 2.0529 (0.5477)	− 1.7567 (0.2852)	− 2.1509 (0.6124)	− 1.9678 (0.2378)	− 2.1495 (0.5877)	− 1.8975 (0.2707)	− 2.1639 (0.6151)
*Period*								
1990	0.9019 (0.0829)	0.0557 (0.3380)	0.6954 (0.1225)	0.0841 (0.4227)	0.8891 (0.0761)	0.1468 (0.3319)	0.6181 (0.1056)	− 0.0491 (0.3987)
1995	− 0.0624 (0.0737)	− 0.2527 (0.2136)	− 0.1458 (0.0944)	− 0.2802 (0.2618)	− 0.0886 (0.0696)	− 0.1876 (0.2100)	− 0.1530 (0.0860)	− 0.1870 (0.2486)
2000	− 0.3627 (0.0671)	− 0.3566 (0.1093)	− 0.5122 (0.0800)	− 0.4127 (0.1205)	− 0.3134 (0.0624)	− 0.3098 (0.1090)	− 0.3745 (0.0724)	− 0.2527 (0.1187)
2005	− 0.2563 (0.0637)	− 0.1006 (0.1070)	− 0.3274 (0.0768)	− 0.2281 (0.1210)	− 0.1801 (0.0582)	− 0.1631 (0.1076)	− 0.1828 (0.0670)	− 0.0087 (0.1160)
2010	− 0.1885 (0.0760)	0.1762 (0.2152)	− 0.0606 (0.0983)	0.2273 (0.2642)	− 0.1704 (0.0698)	− 0.2749 (0.2143)	− 0.0806 (0.0857)	0.1209 (0.2504)
2015	− 0.0321 (0.1013)	0.4780 (0.3451)	0.3505 (0.1350)	0.6095 (0.4279)	− 0.1367 (0.0949)	0.7887 (0.3351)	0.1728 (0.1193)	0.3767 (0.4064)
*Birth cohort*								
1940–1944	− 0.7524 (0.4953)	− 1.7354 (2.4721)	− 1.2019 (0.8175)	− 2.1777 (3.2733)	− 0.6625 (0.4487)	− 1.4954 (2.3632)	− 0.9594 (0.6806)	− 1.8956 (3.0055)
1945–1949	− 0.1425 (0.3123)	− 0.9703 (1.5766)	− 0.3710 (0.5004)	− 0.8736 (1.6753)	− 0.1121 (0.2835)	− 0.8130 (1.5608)	− 0.3928 (0.4389)	− 0.9749 (1.7145)
1950–1954	− 0.0072 (0.2265)	− 0.3966 (1.0631)	0.1401 (0.3337)	0.0176 (1.1198)	− 0.0417 (0.2077)	− 0.5283 (1.1374)	0.1240 (0.2854)	− 0.2420 (1.1555)
1955–1959	− 0.0447 (0.1746)	− 0.1085 (0.7528)	0.2061 (0.2565)	0.3059 (0.8626)	− 0.1052 (0.1598)	− 0.2948 (0.7934)	0.1174 (0.2214)	0.0522 (0.8503)
1960–1964	0.1062 (0.1338)	0.6017 (0.5441)	0.4680 (0.1969)	0.8838 (0.6654)	0.0194 (0.1230)	0.2703 (0.5417)	0.2639 (0.1697)	0.7948 (0.6331)
1965–1969	0.0267 (0.1055)	0.8425 (0.4051)	0.4448 (0.1555)	1.0623 (0.4970)	− 0.1170 (0.0968)	0.6991 (0.3999)	0.1255 (0.1325)	0.8303 (0.4729)
1970–1974	0.4025 (0.0927)	1.1068 (0.2814)	0.7983 (0.1296)	1.2502 (0.3394)	0.2555 (0.0852)	1.0162 (0.2788)	0.4505 (0.1106)	0.8978 (0.3250)
1975–1979	0.4147 (0.0838)	0.9599 (0.1706)	0.6651 (0.1126)	0.9760 (0.1964)	0.3065 (0.0759)	0.9963 (0.1713)	0.4079 (0.0936)	0.7094 (0.1898)
1980–1984	0.2720 (0.0787)	0.6563 (0.1080)	0.3761 (0.1064)	0.5712 (0.1243)	0.1957 (0.0702)	0.8780 (0.1084)	0.3229 (0.0835)	0.5577 (0.1128)
1985–1989	− 0.1476 (0.0938)	− 0.0496 (0.1760)	− 0.3166 (0.1300)	− 0.2827 (0.2258)	− 0.1150 (0.0829)	0.1560 (0.1677)	− 0.1397 (0.1003)	− 0.0499 (0.2013)
1990–1994	− 0.5453 (0.1361)	− 0.7429 (0.3071)	− 0.9911 (0.1958)	− 1.0927 (0.3972)	− 0.3231 (0.1178)	− 0.7372 (0.2921)	− 0.5719 (0.1469)	− 0.6171 (0.3571)
1995–1999	0.4176 (0.3391)	− 0.1639 (0.5337)	− 0.2179 (0.6149)	− 0.6404 (0.8088)	0.6994 (0.2730)	− 0.1472 (0.4802)	0.2517 (0.4067)	− 0.0629 (0.6348)
Intercept	2.7530 (0.0732)	1.3246 (0.3271)	2.1342 (0.1212)	1.2487 (0.4069)	2.9818 (0.0647)	1.1956 (0.3202)	2.5141 (0.0971)	1.2952 (0.3831)
*Model fitting tests*								
DF	20	20	20	20	20	20	20	20
Deviance	35.3641	10.2297	25.5717	12.5918	42.7560	37.2338	35.3089	12.1500
AIC	6.5416	4.9183	5.8856	5.0101	6.8816	5.4642	6.3428	4.8999
BIC	− 39.3892	− 64.5237	− 49.1817	− 62.1616	− 31.9974	− 37.5196	− 39.4445	− 62.6034

Standard errors in brackets

Age effects assume an inverted U-shaped curve. In Fig. 3, fertility increase and then decline by ages, with a peak value at ages 20–24 or 25–29 years, like a normal distribution a little to left. This result is in accordance with those estimated by Fukuda [31] and Kye [32]. The age effects of the national all-order births increase from − 0.92 in age group 15–19 to 1.88 in age group 20–24, and in the same age groups, the age effects of first birth increase from 0.44 to 2.69, respectively, with a higher peak value, and then decrease. If we set the age effect in age group 15–19 to 1, the age effect in age group 20–24 reaches the peak at 16.5, then drops to 0.38 in age group 45–49. This means that women aged 20–24 years have a higher fertility intention than those aged 15–19 years, with the lowest fertility intention at ages 45–49 years. Age effects are associated with timing. These estimates are in accordance with women’s life cycles, in which women mostly attend school at ages 15–19 years, participate in the labor force at ages 20–24 years, balance work and home life at ages 25–29 and 30–34 years, and then experience a downward trend in physical condition after age 40 years. The peak of age effects in cities was in age group 25–29 and not 20–24, compared with that in villages and towns, which means that the peak of fertility intention in cities emerged much later than in villages and towns. The age effects of first birth are similar to those of the all-order births but in the shape of an inverted J. This indicates that age effects at ages 15–19, 20–24, and 25–29 years are much higher that the corresponding age groups in the national sample, which is remarkable at ages 20–24 years. In these three age groups, the age effects in villages are higher than those in cities and are then reversed.

Period effects show a U-shaped curve. The size of period effects first decrease and then increase. This has been negative since 1995 and has continuously dropped to the minimum and then rebounded (it is positive in cities and towns in 2015). This indicates that period effects have contributed to the decline in fertility. If we set the period effect in 1990 to 1, it is 0.38 in 1995 and rises after 2000, from 0.28 in 2000 to 0.39 in 2015. This indicates although fertility policy isn’t relaxed in such a low-fertility situation, there exists a little more intensive fertility intentions. Actually, the problems caused by low fertility and an aging population have forced the birth control policy to be gradually relaxed, and the period effects tend to be stronger with relaxation of the fertility policy. In 2011, couples were allowed to have a second child if both partners were only child, then in 2013, couples were permitted to have a second child if one partner was only child. At the end of 2015, the universal two-child policy was fully implemented. The trends of period effects in cities, villages, and towns are the same, first rising and then falling. The period effects in cities were with a minimum of 0.24 in 2000, but rebounded most intensely. On the contrary, period effects in villages were less affected because fertility policies were loosely implemented. The period effects of first birth are lower, then similar to, then higher since 2005 than those of the national all-order births, which were higher in cities than those in villages during 2005–2015.

As Fig. 3 displays, cohort effects show an inverted U-shaped plus V-shaped curve. Cohort effects first rise, then drop and rebound, which are much more different with age effects and period effects. The cohort effects of the national all-order samples are − 0.7524 in 1940–1944 cohorts, then increase to 0.4147 in 1975–1979 cohorts, and decrease to − 0.5453 in 1990–1994 cohorts. If we set the cohort effects for 1940–1944 cohorts to 1, these increase to 3.21 during 1975–1979 and then decrease to 1.23 during 1990–1994. The cohorts with the most remarkable effects in the national and rural samples are the 1975–1979 cohorts whereas those with the most remarkable ones in cities and towns are the 1970–1974 cohorts, with cohort effects 0.7983 and 0.4505, respectively. The cohort effects of first birth are similar to those of the all-order births, but a little higher in 1960–1964, 1965–1969, 1970–1974, 1975–1979, and 1980–1985 cohorts. These groups experienced different historical events from 1940–1944 cohorts to 1995–1999 cohorts.

### Effects of education on fertility patterns

The results of regression models are displayed in Table 2. Age effects are significant at the 1% level, when the influence of age on fertility is remarkably positive. However, *Age* squared is negative, forming an inverted U-shaped curve. The empirical results accord with the reproductive facts, and there is a nonlinear relationship between fertility and age in which fertility increases with age, then reaches a peak value and declines. Model 3 shows that the inflection point is at ages 26–33 years, in accordance with the data analyzed in Table 1.

**Table 2:** Model estimates

	Model 1	Model 2	Model 3
*Age*	11.17*** (1.962)	8.147*** (2.062)	14.70*** (2.200)
*Age*^*2*^	− 0.213*** (0.030)	− 0.150*** (0.032)	− 0.220*** (0.032)
*Edu*	− 3.193* (1.816)	11.85*** (3.457)	3.899 (3.829)
Periods(taking *P*_*90*_ as base year)			
*P*_*95*_		− 33.17** (14.070)	− 58.83*** (15.676)
*P*_*00*_		− 40.37*** (12.988)	− 73.92*** (16.363)
*P*_*05*_		− 47.34*** (14.611)	− 73.27*** (15.931)
*P*_*10*_		− 53.93*** (14.618)	− 82.67*** (16.375)
*P*_*15*_		− 71.26*** (17.575)	− 87.81*** (16.739)
*Zone*	N/C	C	C
*Pol*	N/C	N/C	C
C	− 51.90 (34.523)	− 116.3*** (34.052)	− 113.1*** (40.262)
Obs	168	168	168
R^2^	0.362	0.414	0.520
F	54.34	18.48	18.52

(1)***p < 0.01, **p < 0.05, *p < 0.1; (2) The robust standard errors are shown in parentheses

The results displayed in Table 2 also show the different influence of education and the birth control policy on the evolution of fertility patterns. Model 1 (no control for any variables) indicates that *Edu* is negative at the 10% level. With controlling for periods and regions (Model 2), R^2^ increases and the coefficient of *Edu* improves to become positive at the 1% level. The inconsistency in the results of Model 1 and Model 2 shows that there may be missing variables. So, in Model 3, we further controlled for special background factors in China, i.e., the fertility policy *Pol*. We find that the increase of R^2^ and *Edu* is no longer statistically significant. This indicates that the effect of education on fertility has not been reflected correctly under the control of the one-child policy. The rigid one-child fertility policy exerted a crowding out effect on education. Even if well-educated women had intense fertility intention, the fertility policy offset their desire to have more children. This finding accords with the actual situation in China. The rigid one-child policy depresses the fertility desire that arises with improvement in SES, living standards, and educational attainment, which accords with the above-analyzed cohort effect. Model 3 indicates that the period effects are negative, which means that the period’s negative effect on fertility is enhanced.

## Discussion

Age is a key factor affecting reproductive physical and physiological conditions, as are period and cohort, considering socioeconomic conditions and historical events in explaining fertility transitions. We analyzed the APC effects of fertility and explained the evolutionary trends in fertility among Chinese women and the characteristics of these trends, together with other factors, such as China’s one-child policy, the improved living conditions resulting from China’s Reform and Opening-up, and the increase in childrearing costs. On the one hand, the fertility policies are very different, from no control to family planning programs and up to the recent policy loosening. On the other hand, the socioeconomic status and educational conditions experienced among women in these cohorts are also remarkably different. Thus, the cohort effects of women born in different eras differ, with era characteristics. One possible reason for this is that women’s fertility intentions intensify with improvement of living conditions, which roughly fits Easterlin’s relative income hypothesis [33, 34]. However, the fertility policy change from encouraging fertility when People’s Republic of China was established in 1949 to controlling births (e.g., the one-child policy which was formally enforced in 1980) has depressed fertility desires resulting from socioeconomic improvement.

Moreover, we examined the effect of improvement in educational attainment among women on fertility transitions. APC effects show temporal changes in fertility over a period of 25 years. In fact, the period effects and cohort effects are related to the fertility policy and reflect the changes across different eras. Given that fertility policies pursue in-time fertility changes rather than economic and social changes, period effects would be much greater than cohort effects. With implementation of the universal two-child policy, the crowding out effect of the fertility policy on education is weakened. Nevertheless, childbearing costs (e.g., opportunity costs, economic pressure, time consuming) would suppress, even offset one’s desire for a second child [35]. More effective measures should be introduced to encourage couples to have a second child. Fiscal, tax, and financial stimuli could help resolve the issue of high childcare costs and enhance period effects and help childbearing-age women balance family and work. In East Asian countries, measures such as tax deduction and exemption (e.g., South Korea), extension of maternity and parental leave (e.g., Singapore, South Korea), increase of children allowance/subsidy (e.g., Japan, Singapore), parents’ re-employment after childbearing (e.g., Japan), more childcare services (e.g., Japan, South Korea) were adopted to encourage fertility [36]. Those measures are worth learning for the sake of China’s policy adjustments. Moreover, the above-mentioned results show that historical events such as economic growth could affect women’s fertility intention. If economic growth can promote improvement in household economic conditions greatly, compared with the childbearing costs, fertility desires would be enhanced.

The year 2015 represents a turning point in the fertility history of China. However, late childbearing is an irreversible trend in China. After implementation of the universal two-child policy, fertility desires were affected, especially at the beginning of this policy. Well-educated or/and older women of childbearing age became more willing to have a second child. Thus, later fertility could rebound greatly. Staggered maternity leave for women of childbearing age should be advocated according to older ages of childbearing. However, later childbearing also means higher reproductive health risks and costs for infant care. Preventing later childbearing or taking measures to encourage women to give birth at the younger ages (but not under legal age) would help to relieve population aging and fertility declining. We advocate the adoption of financial policies to discourage further delays in childbearing. Health services could provide greater support for late-childbearing women to reduce reproductive risks and maintain a balance between family and work. Moreover, we wish that the birth quota would be abolished in the near future, to help China deal with the rapid population aging. Actually, China’s aging is really serious. According to UNDP’s projections, the proportion of population aged 65 and above in China would increase from 9.6% in 2015 to 27.6% in 2050, meanwhile the old-age dependency ratio would increase from 0.13 to 0.47 [37].

### Limitations and future directions

Despite the above-mentioned findings, the study has several limitations. First, the existing statistical data may not be enough for analyzing fertility trends, and more sampling, more longitudinal/tracing data may enhance the persuasiveness of results. Second, due to the cross-sectional design of the study, the effect of education over time is not known, and how education operates during women’s childbearing span should be considered in order to gain more understanding about the relationship between education and fertility. Third, additional new models and methods are needed for testing the influence of historical events on the evolution of fertility trends. Last, the situation in the field of fertility is changing since implementation of China’s two-child policy. According to data of the National Statistical Bureau of China [38], the number of births decreased by 0.58 million and the birth rate decreased by 0.46‰ in 2019, as compared with 2018. The percentage of second children among births was nearly 59.5%, which was 2.1% higher than that in 2018. The first-birth fertility is declining. Therefore, more attention should be given to women’s fertility desire and the trends of fertility postponement, although fertility timing is the result of a complex interplay of environmental and psychophysiological influences. Future research should focus on the underlying mechanisms.

## Conclusions

The period 1990–2015 is a critical phase in the demographic evolution of China and also affects future demographic trends. Based on descriptive analysis and APC accounting models, we provide a summary of temporal effects on fertility trends, which can be delineated into changes across ages, over periods, and among birth cohorts. We revealed the evolutionary trends of fertility patterns among Chinese women using APC analysis of fertility from 1990 to 2015, and found that the fertility patterns are clearly different between urban and rural regions.

First, age effects assume an inverted U-shaped curve. The peak of age effects in cities is in age group 25–29 and not 20–24, compared with that in villages and towns, which means that the peak of fertility intention in cities emerged much later than in villages and towns.

Second, period effects on fertility show a U-shaped curve. The size of period effects first declines and then rises. The trends of period effects in cities are not different from those in villages and towns, first increasing and then declining. The period effects in cities were with a minimum of 0.24 in 2000, but rebounded the most intensely.

Third, cohort effects show an inverted U-shaped plus V-shaped curve. Cohort effects first rise, then drop, and rebound in the end, which are much more different with age effects and period effects. The effect curves of first birth are similar to those of the all-order births, but with different magnitudes, showing remarkable differences not only with respect to age but also period and cohort. The age effects of first birth are much more important in the “Golden Ages” (20–29 years). Period effects enhance, more remarkably in cities than in villages. Cohort effects become much more important after enforcement of the one-child policy.

Last, the influence of education on fertility is not well reflected under the one-child policy. The rigid fertility policy exerted a crowding out effect on education. The analysis of women’s educational attainment shows that even though well-educated women had a strong fertility intention, the rigid birth-control policy offset their desire for more children.

## Data Availability

The datasets supporting the conclusions of this article are included in the tabulations of the National Population Census in 1990, 2000, and 2010, and the 1% National Population Sample Survey in 1995, 2005, and 2015 in China.

## Abbreviations

APC: Age-period-cohort
TFR: Total fertility rate
ASFR: Age-specific fertility rates
SES: Socioeconomic status
IE: Intrinsic estimator
UNDP: The United Nations Development Programme
